# The Influence of Additional Treatments on the Survival of Patients Undergoing Transarterial Radioembolization (TARE)

**DOI:** 10.3390/curroncol31030114

**Published:** 2024-03-13

**Authors:** Natale Quartuccio, Salvatore Ialuna, Daniele Scalisi, Fabio D’Amato, Maria Rosa Barcellona, Maria Grazia Bavetta, Giorgio Fusco, Enrico Bronte, Emma Musso, Fabrizio Bronte, Viviana Picciotto, Antonio Carroccio, Francesco Verderame, Giuseppe Malizia, Angelina Cistaro, Fabio La Gattuta, Antonino Maria Moreci

**Affiliations:** 1 Nuclear Medicine Unit, Ospedali Riuniti Villa Sofia-Cervello, 90146 Palermo, Italy; n.quartuccio@villasofia.it (N.Q.); medicinanucleare@villasofia.it (A.M.M.); 2 Health Physics Unit, Ospedali Riuniti Villa Sofia-Cervello, 90146 Palermo, Italy; fisicosanitario@ospedaliriunitipalermo.it; 3 Unit of Interventional Radiology, Ospedali Riuniti Villa Sofia-Cervello, 90146 Palermo, Italy; f.damato@villasofia.it (F.D.); f.lagattuta@ospedaliriunitipalermo.it (F.L.G.); 4 Internal Medicine Unit, Ospedali Riuniti Villa Sofia-Cervello, 90146 Palermo, Italy; mary.barcellona81@gmail.com (M.R.B.); mariagraziabavetta@gmail.com (M.G.B.); giorgiofusco55@gmail.com (G.F.); viviana.picciotto@ospedaleniguarda.it (V.P.); medicina.cervello@villasofia.it (A.C.); 5 Clinical Oncology Unit, Ospedali Riuniti Villa Sofia-Cervello, 90146 Palermo, Italy; e.bronte@villasofia.it (E.B.); emmamusso@hotmail.it (E.M.); f.verderame@ospedaliriunitipalermo.it (F.V.); 6 Gastroenterology Unit, Ospedali Riuniti Villa Sofia-Cervello, 90146 Palermo, Italy; f.bronte@ospedaliriunitipalermo.it (F.B.); g.malizia@ospedaliriunitipalermo.it (G.M.); 7 Nuclear Medicine Department, Salus Alliance Medical, 16128 Genoa, Italy; angelinacistaro06@gmail.com; 8 AIMN Pediatric Study Group, 20159 Milan, Italy

**Keywords:** transarterial radioembolization, hepatocellular carcinoma, liver metastasis, microspheres, Yttrium-90

## Abstract

The aim of this study was to present our preliminary experience with transarterial radioembolization (TARE) using Yttrium-90 (^90^Y), compare the cancer-specific survival (CSS) of patients with hepatocellular carcinoma (HCC) and colorectal cancer (CRC) liver metastases undergoing TARE, and investigate the influence of additional treatments on CSS. Our database was interrogated to retrieve patients who had undergone TARE using Yttrium-90 (^90^Y) glass or resin microspheres. Kaplan–Meier curves and the log-rank test were employed to conduct survival analysis for the different groups (*p* < 0.05). Thirty-nine patients were retrieved (sex: 27 M, 12 F; mean age: 63.59 ± 15.66 years): twenty-three with hepatocellular carcinoma (HCC) and sixteen with CRC liver metastasis. Globally, the patients with HCC demonstrated a significantly longer CSS than those with CRC liver metastasis (22.64 ± 2.7 vs. 7.21 ± 1.65 months; *p* = 0.014). Among the patients with CRC liver metastasis, those receiving TARE and additional concomitant treatments (n = 10) demonstrated a longer CSS than the CRC patients receiving only TARE (9.97 ± 2.21 vs. 2.59 ± 0.24 months; *p* = 0.06). In the HCC group, there was a trend of a longer CSS in patients (n = 8) receiving TARE and additional treatments (27.89 ± 3.1 vs. 17.69 ± 3.14 months; *p* = 0.15). Patients with HCC seem to achieve a longer survival after TARE compared to patients with CRC liver metastases. In patients with CRC liver metastases, the combination of TARE and additional concomitant treatments may improve survival.

## 1. Introduction

Transarterial radioembolization (TARE) stands at the forefront of modern medical interventions, representing a sophisticated and minimally invasive procedure that uses radioactive microspheres to deliver targeted radiation therapy to liver tumors [1]. The microspheres are predominantly labeled with Yttrium-90 (^90^Y), which is a beta emitter (2.280 MeV (Emax)) radionuclide with a half-life of 64.1 h, ultimately decaying into zirconium-90, with a reasonably high linear energy transfer (LET) and an approximate emission range of 5 mm [2]. The ^90^Y-loaded microspheres are injected into the hepatic artery, the main blood vessel that supplies blood to the liver. This procedure allows the microspheres to be delivered directly to the tumor, minimizing exposure to the surrounding healthy tissues. The specificity of this delivery mechanism is attributed to the short penetration range of β radiation, which travels a short distance before it is absorbed by tissue [3].

In the past decade, there has been growing utilization of TARE, specifically employing either ^90^Y-glass or resin microspheres, particularly in patients affected by hepatocellular carcinoma (HCC) or unresectable colorectal cancer (CRC) liver metastases [4]. Indeed, according to the information that is currently available, TARE is a safe and effective treatment for cancer that has metastasized to the liver as well as HCC.

HCC is still a prevalent and extremely lethal kind of cancer. According to the recent data, the incidence of HCC is increasing globally, with regional differences being noted. Liver cancer claimed 818,000 lives worldwide in 2013, a 9% rise from the 752,000 fatalities reported in 2010 [5,6]. Surgical excision and liver transplantation are the main therapies for hepatocellular carcinoma (HCC). Unfortunately, due to various reasons, such as multiple unresectable tumors or underlying chronic liver disease, only a small fraction (20–30%) of HCC patients are eligible for surgery. With great local tumor control and good survival rates, tumor ablation—which uses procedures like alcohol or acetic acid injection, microwaves, lasers, cryoablation, and the widely utilized radiofrequency—has emerged as a popular and very successful non-surgical option. Its effectiveness, however, decreases for larger tumors. The recommended course of treatment for larger and more advanced-stage malignancies is transarterial chemoembolization (TACE). TACE entails injecting embolic material, such as gelatin sponge particles, after the intra-arterial infusion of lipiodol and a chemotherapeutic drug, such as doxorubicin. Though successful, there are a few contraindications that restrict the tolerability of TACE and make TARE an alternative efficient treatment option [7].

CRC is one of the most common malignancies worldwide, which ranks third in terms of frequency (10.2% of all cancer cases worldwide) and second in terms of being a major cause of cancer-related deaths (9.2% of all cancer deaths worldwide) [8]. With 15–25% of patients with CRC presenting with distant metastases at initial diagnosis, the liver is the major location for distant dissemination [9] in approximately 60% of people who develop metastases [10]. Patients who have liver-dominant metastases and are not candidates for resection or ablation may receive chemotherapy, drug-eluting beads, or radioactive particles directly into the hepatic artery through intraarterial infusion. The goal of intraarterial therapy is to minimize liver toxicity and prevent systemic effects by directly administering a concentrated dosage of chemotherapy or radiation via a catheter into the tumor arterioles. The portal vein supplies most of the blood to the normal liver, whereas the hepatic artery supplies most of the blood to hypervascular metastases (such as neuroendocrine tumors) and hepatocellular carcinomas (HCCs). In comparison to the liver parenchyma, hypovascular metastases—like colorectal cancer—also show a greater hepatic artery supply. In order to achieve specificity in intraarterial therapy, the catheter must be strategically positioned, and the arterial flow must be directed preferentially toward the tumor [11,12].

Regarding the choice between glass and resin microspheres for TARE, glass microspheres offer a distinct advantage of delivering a specific radiation dose with a reduced number of particles, which can potentially minimize embolic effects. Therefore, they are considered a more appropriate treatment option in situations where achieving early stasis or addressing reflux is a concern, especially in cases of HCC with portal vein invasion and for radiation segmentectomy. In contrast, resin microspheres have a lower activity per particle, necessitating a higher number of particles to achieve the same radiation dose. As a result, resin microspheres are typically preferred for larger tumors and those with a high arterial flow [13].

TARE has been extensively proved as an effective first- or second-line treatment in patients with advanced HCC. In contrast to the conventional treatment protocols for the intermediate and advanced stages, namely transarterial embolization and sorafenib, radioembolization consistently yields comparable survival rates [14]. Furthermore, the RESIN trial demonstrated in a multicenter cohort of 498 patients that TARE is an effective second-line treatment for patients with CRC liver metastases [4].

Overall, although there is mounting evidence with regards to the beneficial effect of TARE in HCC and CRC liver metastases, there are still limited medical centers with personnel trained to perform TARE. Furthermore, the combined use of systemic therapy and TARE is still debated. Systemic therapy remains the primary recommended treatment for hepatocellular carcinoma (HCC) characterized by macrovascular invasion (MVI). Since sorafenib is the only approved systemic medication for treating advanced HCC, its historical value cannot be denied. Nevertheless, in patients with intermediate-stage illness, the combination of sorafenib with catheter-based therapy has not shown any proven benefits [15,16,17,18,19,20]. Also, a recent meta-analysis did not favor the combined use of sorafenib and TARE in HCC patients [21].

The aim of this study was to compare the effect of TARE on the survival of patients with HCC and patients with CRC liver metastases and to investigate the influence of additional treatments on CSS.

## 2. Materials and Methods

### 2.1. Patients

The database of our center was interrogated by two researchers (N.Q., S.I) to retrieve patients who had undergone TARE with ^90^Y glass or resin microspheres. The eligibility criteria for enrolment in the study were age ≥ 18 years; histologically proven or imaging-based diagnosis of unresectable HCC or CRC liver metastasis with unilobar or bilobar involvement; life expectancy > 6 months; preserved liver function with Child–Pugh Class A or B; Eastern Cooperative Oncology Group (ECOG) performance status ≤ 2; bilirubin < 2.0 mg/dL; albumin > 2.0 g/dL; availability of pre-treatment hepatic angioscintigraphy with ^99m^Tc-macroaggregated albumin (MAA); a post-radioembolization ^90^Y PET/CT scan carried out within 12 h from TARE; information on additional treatment beyond TARE; and a minimum follow-up of 24 months. Patients with CRC liver metastases had undergone surgical removal of the primary tumor before undergoing TARE.

The main exclusion criteria included previous radiotherapy to the liver, ascites, unresolved toxicity from first-line therapy, extra-hepatic metastases, or contraindications to angiography.

For each patient, beyond the inclusion criteria, the following information were retrieved: age, sex, intent (segmentectomy, lobectomy, bridging or palliative care), additional treatment, number of liver metastatic sites (for the CRC group), lobar involvement (uni- or bilobar), diameter of the main tumor lesion within the liver, lobar volume (cm^3^), organ volume (cm^3^), tumor volume (cm^3^), injected activity (GBq), dose to the tumor (GBq), follow-up period (months), side effects and radiation-induced effects, and date of cancer-specific death.

Ethical approval for conducting the study was waived by the local ethics committee in view of the retrospective nature of the study.

### 2.2. Pre-Treatment Imaging

Before TARE, all the patients underwent at our center an angiography of the hepatic vasculature for treatment planning and a liver angioscintigraphy with ^99m^Tc-MAA (fixed dose: 185 MBq) to evaluate the percentage of injected activity shunted to the lungs and foresee the distribution of the ^90^Y-TARE spheres. These procedures were performed in a fully equipped Innova angiography suite (General Electric Healthcare, Milwaukee, WI, USA).

The ^99m^Tc-MAA scan was acquired using a dual-head gamma camera (Infinia; General Electric Healthcare, Milwaukee, WI, USA) and a high-resolution, low-energy collimator. Whole-body anterior and posterior projections of the superior abdominal regions were obtained using 120 s planar pictures, with the energy window set at 140  ±  10 keV. Ninety-nine step-and-shoot mode projections were recorded in 360 degrees (10 s per step) for the SPECT/CT using a 256 × 256 matrix. Each ^99m^Tc-MAA scan was reviewed jointly by two board-certified nuclear medicine physicians, both with >5 years of experience on a Xeleris 1.123 GE workstation (GE Medical Systems, Milwaukee, WI, USA). The CT and ^99m^Tc-MAA SPECT/CT scans were analyzed and compared jointly by an interventional radiologist and a nuclear medicine physician to evaluate the uptake of ^99m^Tc-MAA within the tumor area (CT-MAA agreement) and classified as positive or negative for the presence of shunts to the lungs. In the case of no evidence of a significant hepato-pulmonary shunt fraction (<20%), patients were submitted to ^90^Y-TARE within 21 days from the ^99m^Tc-MAA scan.

### 2.3. The ^90^Y-TARE Procedure

All patients were treated with TARE after multidisciplinary team discussions according to the different intents. The pre-procedural exams for the completion of staging included baseline imaging studies: liver sonography, clinical and laboratory examination, contrast-enhanced CT (ce-CT), and [^18^F]FDG PET/CT. Dosimetric estimations at the voxel level were obtained using the commercially available software Simplicit^90^Y^TM^ v2.4 (Mirada Medical, Oxford, UK). The prescribed ^90^Y activity for the SIR microspheres was determined on the basis of a multicompartment dosimetric estimation, whereas a noncompartmental method was used for the TheraSpheres.

Notably, in the case of the glass microspheres, in the patients with HCC, the absorbed doses were calculated in order to achieve a desirable minimal dose of 120 Gy to the perfused liver for lobar treatment and a minimum tumor-absorbed dose of 205 Gy, as recommended by Salem et al. [22].

After selective catheterization of the right/left hepatic artery to assess the vascular and tumor anatomy and blood flow, the patient was administered with either ^90^Y resin spheres (SIR-Spheres; Sirtex Medical, Sydney, Australia) or ^90^Y glass microspheres (TheraSpheres; Boston Scientific, Boston, MA, USA). The choice of radiopharmaceutical between the ^90^Y resin spheres and ^90^Y glass microspheres was made based on multidisciplinary discussions among the nuclear medicine physician, the interventional radiologist, and the physicist.

### 2.4. Post-Treatment Imaging

Immediately after the ^90^Y-TARE procedure, all subjects underwent a ^90^Y-PET/CT scan within 4–12 h to assess the microsphere distribution pattern, confirm technical success, and detect any non-target activity. The PET/CT scan was carried out using a PET/CT scanner (GE Discovery STE, GE Healthcare, Milwaukee, WI, USA). The first procedure was a CT scan (140 kVp, 30–300 mA, 3.75 mm slice thickness) used for attenuation correction and anatomical localization. A maximum of one or two bed positions was used during the PET scan acquisition, allowing for 15 min per bed position to cover the entire liver. Three iterations and eighteen subsets were used in the reconstruction process.

The ^99m^Tc-MAA SPECT/CT and ^90^Y PECT/CT scans were compared by the same nuclear medicine physician who planned the treatment to evaluate the overlap of the distribution between the MAA particles and microspheres.

### 2.5. Statistics

Categorical variables were characterized by number (%) representation, while continuous data were described using medians and ranges or means ± standard deviation. The Chi-square test was employed to evaluate distinctions between categorical variables, and a two-tailed Student’s *t*-test was applied to assess any difference between two independent patient groups (*p* < 0.05). The distribution of the data was assessed by using the Shapiro–Wilk normality test.

The CSS of each patient was retrieved and measured in months based on the time intercurred from the date of TARE and the date of death at the last available follow-up. Patients who died from causes unrelated to oncological disease were not counted in this measurement. Kaplan–Meier curves were employed to conduct, separately, survival analysis for each group; the survival curves of the different groups were compared using the log-rank test at a 0.05 level of significance (*p* < 0.05). The CSS of the HCC patients was compared with that of the patients with CRC liver metastases. Additionally, for each pathology-based patient group, the influence of additional treatments was assessed, comparing the CSS of patients receiving only TARE with that of patients receiving TARE and additional treatments.

The statistics were calculated using MedCalc 11.3.8.0 (MedCalc software, Mariakerke, Belgium).

## 3. Results

From a total of 150 patients who had undergone TARE, 39 patients with complete datasets (liver angioscintigraphy, post-radioembolization ^90^Y PET/CT scan, and clinical information listed in the eligibility criteria) were retrieved (sex: 27 M, 12 F; mean age: 63.59 ± 15.66 years; 23 with unrespectable HCC and 16 with liver metastases from CRC).

The median follow-up from TARE to the last available record (April 2023) was 69 months (range: 39–91 months). In the HCC group, the indications were segmentectomy (n = 18), lobectomy (3), sequential lobectomy (1), and bridging (1). In the CRC group, the indications were segmentectomy (n = 6), lobectomy (4), sequential lobectomy (2), and palliative care (4).

The mean administered activity was 2.27 GBq, and the mean dose to the tumor was 257.56 Gy. A total of 10 of 16 patients with liver metastases from CRC received additional treatments with sorafenib (n = 7), regorafenib (n = 2), or both (n = 1). Eight patients in the HCC group received additional treatments (sorafenib).

Regarding the safety of TARE, in the HCC group, 15 cases of side-effects were registered: pain, nausea, ascites, jaundice, dyspnea/pleural effusion, asthenia, pruritus, portosystemic encephalopathy. A total of 3 of these 15 patients also experienced radiation-induced side effects, including radiation-induced liver disease (n = 2) and actinic pneumonia (n = 1). In the CRC group, 9/16 patients experienced side effects including fever, pain, nausea, ascites, dyspnea/pleural effusion, cholecystitis, asthenia, and pruritus.

No statistically significant different was found between the categorical and continuous clinical variables of the HCC and CRC patient groups.

A summary of the main results for the whole cohort and the HCC and CRC subgroups is provided in Table 1.

**Figure 1:**
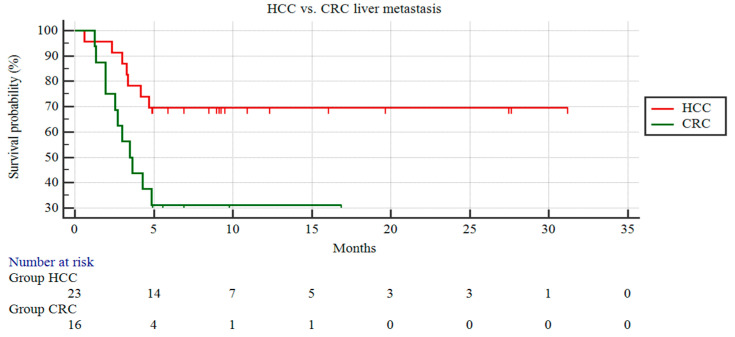
Kaplan–Meier curves of patients with HCC and patients with CRC liver metastases.

The patients with HCC demonstrated a significantly longer CSS than those with liver metastasis from CRC (22.64 ± 2.7 vs. 7.21 ± 1.65 months; *p* = 0.014) (Figure 1).

### 3.1. Patients with HCC

**Figure 2:**
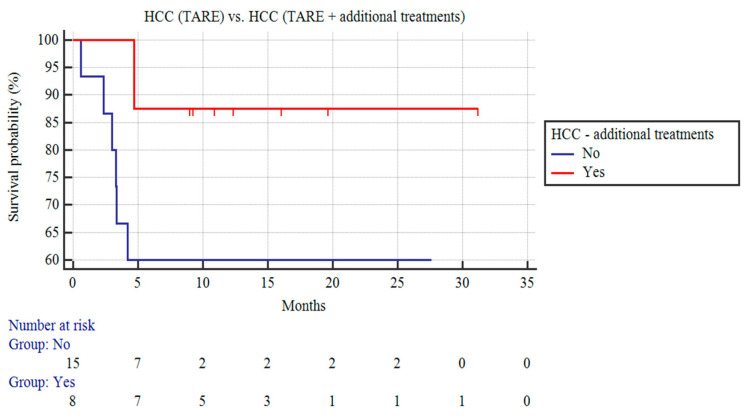
Kaplan–Meier curves of patients with HCC undergoing TARE and patients with HCC receiving TARE and additional treatments.

Among the patients with HCC, the patients receiving TARE and additional treatment (n = 8) demonstrated a trend of a longer CSS than the HCC patients receiving only TARE, although this did not reach statistical significance (n = 15; 27.89 ± 3.1 vs. 17.69 ± 3.14 months; *p* = 0.15; Figure 2).

### 3.2. Patients with CRC Liver Metastases

**Figure 3:**
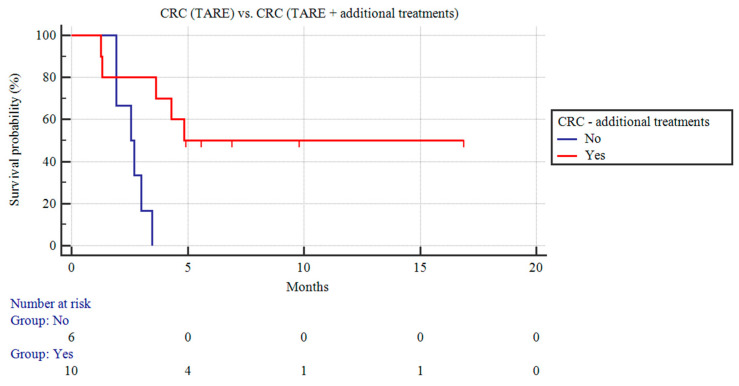
Kaplan–Meier curves of patients with CRC liver metastases undergoing TARE and patients with CRC liver metastases receiving TARE and additional treatments.

Among the patients with CRC liver metastases, those receiving TARE and additional concomitant treatments (n = 10) demonstrated a longer CSS than the CRC patients receiving only TARE (9.97 ± 2.21 vs. 2.59 ± 0.24 months; *p* = 0.06; Figure 3).

**Figure 4:**
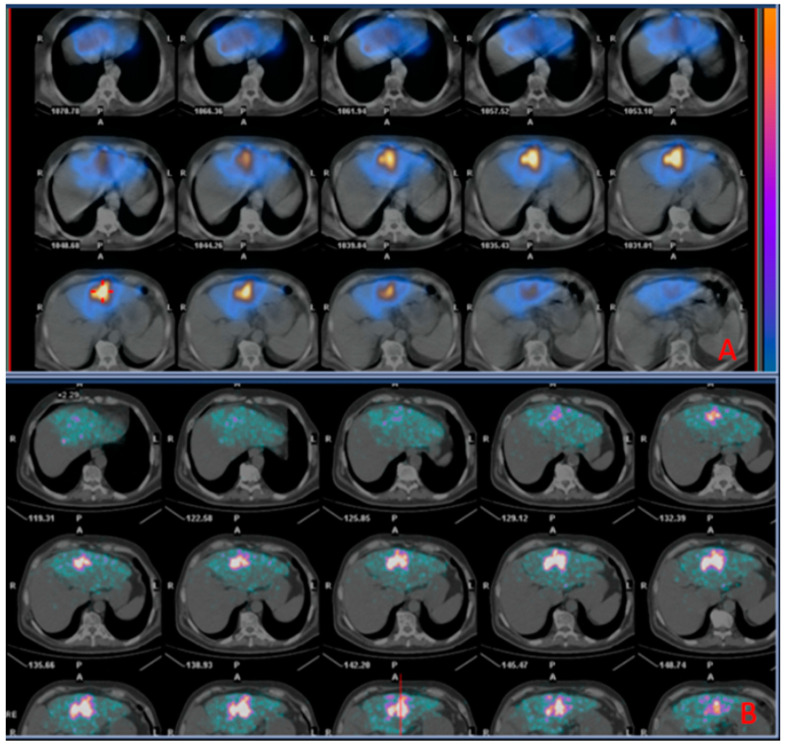
Images of a patient undergoing radioembolization of a hepatic metastasis from colorectal cancer. (**A**) (Upper box): SPECT/CT images (diagnostic phase) 2 h acquired after administration of ^99m^Tc-MAA; (**B**) (Inferior box): PET/CT images (therapeutic phase) acquired 2 h after the administration of ^90^Y TheraSpheres, showing successful distribution of ^90^Y TheraSpheres matching that of ^99m^Tc-MAA in the diagnostic phase.

A representative case of a patient with CRC liver metastasis treated with TARE is shown in Figure 4.

## 4. Discussion

TARE is an effective therapy that can be used with different intents. It can be used to shrink tumors before surgery or liver transplantation. It is also a bridging and downstaging therapy, meaning that it can help patients who are not yet eligible for surgery or transplantation to become eligible [23]. TARE has been traditionally used to treat advanced HCC, but recent improvements in the technique have made it effective for treating solitary HCC as well. Furthermore, it is a safe procedure when performed in the context of an expert multidisciplinary team. Alternative embolization treatments include conventional transarterial chemoembolization (cTACE) and drug-eluting beads (DEB-TACE) [10]. TARE has significantly less complications than conventional transarterial chemoembolization (cTACE) and drug-eluting beads (DEB-TACE) in patients with HCC [24].

The frequency of advanced-stage hepatocellular carcinoma (HCC) with poor prognoses is still on the rise, even with the use of antiviral drugs and improved surveillance systems. For the past ten years, systemic treatment with multitargeted tyrosine kinase inhibitors (TKIs), such as sorafenib, has been the standard approach. Furthermore, TKI combination therapy with other drugs, including as antiangiogenic inhibitors and immune checkpoint inhibitors (ICIs), has shown promise in the treatment of advanced HCC [25]. However, the shortcomings of low disease response rates, unacceptable adverse effects, and a comparatively short overall survival call for the creation of innovative or improved treatments for advanced HCC. Within the boundaries of the intermediate and advanced phases of HCC, two particular scenarios seem especially appealing for the application of TARE. First, patients in between the intermediate and advanced stages are treated. These patients include those with advanced-stage single tumors infiltrating a segmental or lobar branch of the portal vein, as well as those with substantial or bilobar disease who are considered unsuitable candidates for transarterial chemoembolization (TACE). Second, in cases where downstaging could potentially open the door to a more radical treatment strategy, TARE can be taken into consideration for patients who just barely meet the requirements for resection, ablation, or transplantation. Moreover, TARE can be used to treat patients who do not respond well to TACE or sorafenib therapy [14].

For patients with liver-dominant CRC that are not responsive to resection or ablation, radioembolization is advised in accordance with the guidelines from the National Comprehensive Cancer Network. It is also recommended for HCC, or hepatocellular cancer. For colorectal liver metastases that are resistant to chemotherapy, the European Society for Medical Oncology recommend considering radioembolization, especially in salvage circumstances [26]. The current guidelines, however, do not agree on the use of radioembolization as a first-line therapy because there are not enough data. Surgical removal of CRC liver metastases has shown promising 5-year survival rates of 20–70%, making it the preferred treatment option for suitable patients. However, due to technical constraints and the severity of the disease, a significant portion (70–80%) of individuals with extensive liver metastases are unable to undergo surgery [27]. There is expanding evidence that TARE provides a longer time to progression compared to transarterial chemoembolization (TACE) in patients with HCC [28]. Similarly, in patients with CRC liver metastases, according to the results of the EPOCH trial, including 428 patients, TARE reduces the risk of disease progression or death compared to chemotherapy alone [10].

In our center’s experience, patients undergoing TARE with either resin or glass ^90^Y-spheres showed a CSS comparable with that reported in the literature (22.64 ± 2.7 for patients with HCC and 7.21 ± 1.65 months for patients with liver metastases from CRC). Interestingly, the effect of additional treatments had more beneficial results in the group of patients with liver metastases from CRC (CS: 9.97 ± 2.21 months) compared to the patients not receiving concomitant pharmacological treatment (CSS: 2.59 ± 0.24 months). The additional treatments in our CRC patient cohort included sorafenib, regorafenib, or a combination of both. Given the existence of a statistical trend, we suppose that the lack of statistical significance for a longer CSS in HCC patients receiving additional treatments compared to patients receiving only TARE may be attributed to the limited number of the patient sample.

Person-related predictors of unfavorable outcomes in patients undergoing TARE include an older age (>70 years) [29,30], male sex [31], a higher tumor grade, a larger tumor size, a later tumor stage, a lack of treatment with surgery or systemic therapy, the presence of lymphatic or vascular invasion [32], a Child–Pugh score (CPS) of B or C vs. A, and an Eastern Cooperative Oncology Group performance status (ECOG-PS) of 2 or 1 vs. 0 [33,34]. Other factors that positively influence survival in patients receiving TARE are administered activity and mean dose to the tumor [35]. Further, the prospective CIRT study demonstrated as independent prognostic factors for OS in patients with HCC treated with TARE the presence of ascites, right-sided tumors, the presence of portal vein thrombosis, and main portal vein thrombosis, ALBI grades 2 and 3 [34].

Side effects were reported in a large portion of our patients, in 15/23 (65%) HCC patients and 9/16 (56%) CRC patients. Nevertheless, the majority of these adverse events were not severe, confirming the safety profile of TARE.

Our study had several limitations. First, we have to mention the limited sample size of the samples. Furthermore, different groups received different protocols of systemic therapy and different intents behind TARE. The limited sample size and the numerous clinical variables might have influenced the individual CSS in our patient cohort, and our findings would need to be reassessed in studies with larger patient samples.

Future studies may assess whether the combination of TARE and systemic therapy may improve survival in other cancers, such as cholangiocarcinoma and other oncological diseases metastasizing to the liver, such as breast cancer and neuroendocrine tumors. Other investigations may also assess the additional effect on survival of immunotherapy in HCC and CRC patients treated with TARE. Furthermore, the potential benefit of using jolmium-166 (^166^Ho) as a radionuclide for TARE may be examined. Indeed, due to its imaging properties, specialists have been interested in the therapeutic isotope ^166^Ho since it was first suggested as a possible option for liver radioembolization therapy. Indeed, ^166^Ho is a beta emitter that also belongs to the lanthanide group and emits gamma rays. As a result, it may be detected using magnetic resonance imaging and single-photon emission computed tomography, respectively. The capacity to carry out the scout and treatment processes with a single particle is another benefit of ^166^Ho. This provides more control over dosimetry-based patient selection and treatment planning and opens the door to the potential for a customized treatment strategy [36].

The use of machine learning and radiomics in the treatment of malignant liver tumors may greatly change medical procedures in future. Radiomics uses sophisticated algorithms to extract various quantitative aspects, such as texture, intensity, and structure, from medical images. Deep learning in particular, which is a type of machine learning, makes it easier to integrate and analyze these data from various picture sources. This potent combination makes it possible to forecast how malignant liver tumors will react to various treatments and situations [37].

## 5. Conclusions

Patients with liver metastases from CRC receiving TARE had a shorter survival than those with HCC. In patients with liver metastases from CRC, additional concomitant treatments and increasing the injected activity and mean dose to the tumor are recommended in order to achieve a better outcome.

## Figures and Tables

**Table 1:** Main characteristics of the HCC and CRC patient groups.

	Whole Cohort	HCC	CRC Liver Metastases	*p* Significance
Number of patients	39	23	16	NA
Mean age (SD), years	63.59 ± 15.66	66.77 ± 13.67	58.60 ± 17.46	ns
Sex (m)	27	17 (73.9%)	10 (62.5%)	ns
ECOG performance status 1	7	4	3	ns
Palliative intent of TARE (n. patients)	4	0	4	ns
Patients receiving additional treatments	18 (46.1%)	8 (34.7%)	10 (62.5%)	ns
Mean number of liver metastatic sites	2.1 ± 0.6	2.17 ± 0.8	2	ns
Bilobar involvement (n. patients)	12	4 (17.39%)	8 (50%)	ns
Mean diameter of the main tumor lesion	54.32	56.82	49.75	ns
Mean lobar volume (cm^3^)	997.55	942.74	1067	ns
Mean organ volume (cm^3^)	1650.75	1648.33	1653.67	ns
Mean tumor volume (cm^3^)	339.13	360.64	305.67	ns
Mean injected activity (GBq)	2.27	1.98 ± 0.75	2.68 ± 1.01	ns
Mean dose to the tumor (Gy)	257.56	246.93	274.11	ns
Mean follow-up (SD), months	69 (39–91)	69.07 ± 12.07	74.05 ± 6.95	ns
Side effects (n. patients)	23	15 (65.21%)	9 (56.25%)	ns
Radiation-induced side effects (n. patients)	3	3 (13%)	0	NA

## Data Availability

Data can be provided upon reasonable request bona fide.
